# Letter regarding Griffiths *et al.* “anti-Müllerian hormone decreased in women with superficial peritoneal endometriosis and associated with an elevated inflammatory profile”

**DOI:** 10.1530/RAF-26-0127

**Published:** 2026-08-14

**Authors:** Jasper Verguts, Tanne Daniels

**Affiliations:** ^1^ Department of Gynecology and Obstetrics, Jessa Hospital, Hasselt, Belgium; ^2^ UHasselt, Faculty of Medicine and Life Sciences, LCRC(-MHU), Diepenbeek, Belgium; ^3^ Gynecology and Obstetrics, Department of Benign Gynecology, Maastricht University Medical Centre, Maastricht, The Netherlands

**Keywords:** endometriosis, AMH, surgery

Dear Editor,

Griffiths *et al.* report reduced serum AMH and a distinct pro-inflammatory signature in women with isolated superficial peritoneal endometriosis (SPE), independent of any endometrioma (Griffiths *et al.* 2026). Most of the ovarian reserve literature in endometriosis has focused on endometriomas, so this is a genuinely new observation. It also raises a question the cross-sectional design cannot settle: what happens to AMH once these SPE lesions are removed?

Every study the authors cite on post-surgical AMH decline (Wang *et al.* 2020; Muraoka *et al.* 2021; Sarbazi *et al.* 2021; Fakehi *et al.* 2022; Mansouri *et al.* 2022; Shi *et al.* 2022; Tang & Li 2022; Crestani *et al.* 2023) is built around endometrioma cystectomy, where mechanical or thermal follicle loss during cyst wall dissection is usually taken to be the main driver of AMH decline. Guidelines recommend excising any accompanying superficial lesions at the same operation (NICE 2017; Becker *et al.* 2022), so it seems unlikely that any of these cohorts separated that surgical trauma from whatever smaller effect comes from removing SPE at the same time. Given that SPE itself appears to sustain a pro-inflammatory state that suppresses AMH, resolving that inflammatory component could partially offset the cystectomy-related decline – or it might not; the two mechanisms operate at different scales and there is no strong reason to assume they cancel out. Either way, it is a testable question that does not appear to have been asked directly.

The reverse case may be easier to study cleanly and is just as informative. In women with SPE and no endometrioma, does removing the lesions bring AMH back up, in line with the inflammatory story told here? Or does operating near the ovarian fossa and utero-ovarian ligament – diathermy spread, for instance – reduce AMH regardless of any anti-inflammatory benefit? The one existing AMH study in an SPE cohort, Lessans *et al.* (2024), was cross-sectional too, at least from what is described here, and does not report postoperative values.

Griffiths *et al.* (2026) mention an observational cohort and biobank running since 2015, which could support this kind of follow-up. SPE-only surgery versus expectant management would be a reasonably clean prospective comparison, since both are already routine clinical choices. The combined case is messier: guidelines already recommend excising SPE alongside an endometrioma, so a true prospective arm without excision is hard to justify on ethical grounds, and this would more realistically have to rely on retrospective cases where lesions were left *in situ* for other clinical reasons. Any of these would also need to stratify postoperative AMH by subsequent hormone use, since that is what determined whether an effect was seen at all in the present study. This is not just a design detail – it is close to what a clinician needs to know before advising a woman with SPE, and no other apparent fertility problems, on whether surgery will help, harm, or make no difference to her ovarian reserve.
